# Obstructing Sleep Apnea in Children with Genetic Disorders—A Special Need for Early Multidisciplinary Diagnosis and Treatment

**DOI:** 10.3390/jcm10102156

**Published:** 2021-05-17

**Authors:** Mihaela Oros, Lucica Baranga, Vasilica Plaiasu, Sebastian R. Cozma, Adriana Neagos, Luminita Paduraru, Violeta Necula, Cristian Martu, Lucia Corina Dima-Cozma, Dan Cristian Gheorghe

**Affiliations:** 1 Faculty of Medicine, Titu Maiorescu University, 040441 Bucharest, Romania; mihaelaoros70@yahoo.com; 2 Ponderas Academic Hospital, 014142 Bucharest, Romania; barangalucica@yahoo.com; 3 Regional Center of Medical Genetics, INSMC Alessandrescu-Rusescu, 020395 Bucharest, Romania; vasilica.plaiasu@gmail.com; 4 Department of Surgery (II), Faculty of Medicine, Grigore T. Popa University of Medicine and Pharmacy, 700115 Iaşi, Romania; cristimartu@gmail.com; 5 Department of Otorhinolaryngology, George Emil Palade University of Medicine, Pharmacy, Science and Technology, 540142 Tirgu Mures, Romania; neagos.adriana@gmail.com; 6 Department of Mother and Child Care, Faculty of Medicine, Grigore T. Popa University of Medicine and Pharmacy, 700115 Iaşi, Romania; luminita.paduraru@gmail.com; 7 Cuza-Voda Clinical Hospital of Obstetrics and Gynecology, 700038 Iaşi, Romania; 8 Department of Otorhinolaryngology, Faculty of Medicine, Iuliu Haţieganu University of Medicine and Pharmacy, 400000 Cluj-Napoca, Romania; 9 Department of Internal Medicine, Faculty of Medicine, Grigore T. Popa University of Medicine and Pharmacy, 700115 Iaşi, Romania; cdimacozma@yahoo.com; 10 ENT Department “MS Curie” Hospital Bucharest, “Carol Davila” University of Medicine, 050474 Bucharest, Romania; gheorghe.dancristian@gmail.com

**Keywords:** obstructive sleep apnea (OSA), non-invasive ventilation (NIV), polysomnography (PSG), children, genetic disorders

## Abstract

Background—Children with genetic disorders have multiple anatomical and physiological conditions that predispose them to obstructive sleep apnea syndrome (OSAS). They should have priority access to polysomnography (PSG) before establishing their therapeutic protocol. We analyzed the prevalence and the severity of OSAS in a particular group of children with genetic disorders and strengthened their need for a multidisciplinary diagnosis and adapted management. Methods—The retrospective analysis included children with genetic impairments and sleep disturbances that were referred for polysomnography. We collected respiratory parameters from sleep studies: apnea–hypopnea index (AHI), SatO_2_ nadir, end-tidal CO_2_, and transcutaneous CO_2_. Subsequent management included non-invasive ventilation (NIV) or otorhinolaryngological (ENT) surgery of the upper airway. Results—We identified 108 patients with neuromuscular disorders or multiple congenital anomalies. OSAS was present in 87 patients (80.5%), 3 of whom received CPAP, 32 needed another form of NIV during sleep, and 15 patients were referred for ENT surgery. The post-therapeutic follow-up PSG parameters confirmed the success of the treatment. Conclusions—The upper airway obstruction diagnostics and management for children with complex genetic diseases need a multidisciplinary approach. Early detection and treatment of sleep-disordered breathing in children with genetic disorders is a priority for improving their quality of life.

## 1. Introduction

Obstructive sleep apnea syndrome (OSAS) is a disease that has been extensively studied over the few last decades and classified according to its pathogenesis and severity in adults and children. It is defined as sleep-disordered breathing (SDB) that is characterized by prolonged partial upper airway obstruction and/or intermittent complete obstruction (obstructive apnea) that disrupts normal ventilation during sleep and disturbs normal sleep patterns [1,2]. Although obstruction of the upper airway during sleep remains its main feature [3], other symptoms are common in clinical practice, such as frequent snoring, sleep enuresis, headaches on awakening, daytime sleepiness, and attention-deficit and learning problems [1,2]. Physical examination may reveal characteristic signs, such as being underweight or overweight, tonsillar hypertrophy, adenoidal facies, micrognathia/retrognathia, or a high-arched palate [1,2]. Nocturnal hypoventilation may produce nocturnal arousals because of hypoxemia or hypercarbia, awkward sleeping positions, night sweats, morning headaches, irritability, and other symptoms [4,5].

The disease affects 1–10% of children in certain populations [2,6]. The prevalence is differently reported when considering variate age groups, gender, and social and genetic factors [2,7]. Genetic disorders are a category of diseases that include certain types of birth defects, chronic diseases, developmental problems, and sensory deficits, and could appear de novo or are inherited from one or both parents. It is estimated that 1 in 25 children is affected by a genetic disorder [8].

The presence of congenital craniofacial anomalies can be associated with a higher incidence of obstructive sleep apnea than in the regular pediatric population [9]. In these children with congenital malformations or genetic disorders, which are the main cause of OSAS, SDB is not always recognized and reported by the parents [10]. Some studies have shown some degree of association between parental concern and severity of the disease [11].

The connection between craniofacial anomalies and sleep apnea could reside in the anatomic obstruction of the upper airways, which these patients sometimes present. Mucopolysaccharidoses, Down syndrome, muscular dystrophies, and other neurologic disorders have been associated with obstructive sleep apnea [12,13,14]. Impaired sleep is a frequent problem in these subjects and it is commonly either missed or underestimated. The severity of the disease is usually classified according to existing guidelines [15].

Unrecognized OSAS in children may lead to complications whose severity is even more important as OSAS evolves over time [2]. Intermittent hypoxia has been demonstrated even in preterm neonates and severe apnea/bradycardia in infants [16]. Long-term sleep disturbances can produce cognitive impairment, attention-deficit/hyperactivity disorders, and social disabilities that could prove challenging to cope with [2,5,6]. It is difficult to decide which factor contributes the most to long-term cognition complications of OSAS.

Due to the complexity of comorbidities, patients with genetic syndromes frequently require a multidisciplinary approach for both the diagnosis and therapeutic interventions [17]. The management of obstructive sleep-disordered breathing can be surgical or conservative. A tonsillectomy is one of the most used procedures for improving airway patency in children [2,18,19]. Continuous positive airway pressure (CPAP) or other forms of non-invasive ventilation (NIV) are appropriate therapeutic approaches when indicated [20,21]. CPAP is very helpful in OSA since it improves the quality of sleep and alleviates daytime sleepiness and caregiver concern [16,21,22]. Bilevel positive airway pressure (BiPAP) is preferred when there is associated hypoventilation, which involves a complex set of sleep-disordered breathing mechanisms: hypotonia, craniofacial anomalies, obesity, and patients with genetic disorders [23]. For these patients, the benefits from NIV could be different from otherwise healthy children and are focused on the palliation of symptoms and quality of life improvement, both for children and their families [16,24]. Weight loss and intranasal corticosteroids are also management options for particular cases [1]. In neurologically impaired pediatric patients, different therapeutic protocols can be discussed.

The aim of this research was to analyze the prevalence and severity of OSAS in a peculiar group of children with complex neurological, musculoskeletal, and genetic disorders to show that sleep-disordered breathing frequently affects this particular population and to argue for the need for a multidisciplinary approach in the diagnosis and therapeutic management of OSAS that is adapted to the special needs of cases with various genetic diseases.

## 2. Materials and Methods

The present retrospective study was conducted on children that were diagnosed with neurological, musculoskeletal, and complex genetic syndromes who were referred from August 2017 to March 2020 to the pediatric sleep unit of Regina Maria Clinics and Ponderas Academic Hospital Bucharest for complete sleep studies using polysomnography (PSG). For children diagnosed with OSAS with indications for surgical treatment, it was performed at the M.S. Curie Hospital. The protocols for diagnosis and subsequent management were discussed with all the specialists involved and were approved by the Ethics Committee of Ponderas Academic Hospital (no. 245b/05/10/2020). All subjects gave their informed consent for inclusion before they participated in the study and the study was conducted in accordance with the Declaration of Helsinki.

Inclusion criteria were as follows: age under 18 years and the presence of at least one neurological, neuromuscular, or genetic comorbidity. Patients with an uncertain diagnosis or with traumatic or iatrogenic lesions of the upper airway were excluded.

The overnight video PSG was performed using System Alice 6 LDx, Phillips Respironics, with Sleepware G3 software. Transcutaneous CO_2_ (TcCO_2_) values were obtained over the entire PSG recording period using the Radiometer Monitor system.

Respiratory parameters, including apnea–hypopnea index (AHI) and sleep stages, were scored according to the AASM 2012 guidelines [15]. AHI is the sum of apneas plus hypopneas per hour of sleep (events/h).

The OSAS was defined as follows: mild for 1 < AHI ≤ 5, moderate for 5 < AHI ≤ 10, and severe for AHI > 10, with oxygen desaturation index > 3%.

Hypoventilation was defined at TcCO_2_ > 50 mmHg for >25% of the total sleep time (TST) according to guidelines [15].

In this study, non-invasive ventilation was defined as any form of ventilatory support whenever CPAP or BiPAP was applied.

CPAP was chosen in the presence of isolated OSAS and BiPAP was selected in the presence of OSAS in neuromuscular patients or with associated hypoventilation. In all patients, NIV was started in the pediatric respiratory ward or the pediatric sleep laboratory. The final goal was achieving uninterrupted supine rapid eye movement (REM) sleep by manually selecting the NIV pressure [25]. The pressures were titrated according to international guidelines until the complete elimination of respiratory events and the normalization of pulse oximetry and TcCO_2_ with good sleep efficiency (SE) [25]. The patients and their parents were trained on how to use ventilatory masks and how to prevent adverse effects. In children with an obvious clinical anatomical obstacle (adenoid vegetations, chronic tonsillar hypertrophy), surgical treatment of the obstruction was proposed.

The following parameters were collected for the study: the presence or absence of different symptoms of OSAS, the results of the clinical otorhinolaryngological examinations, polysomnographic studies data, CPAP or BiPAP parameters, and complications associated with the NIV. Endoscopic evaluations were also recorded when available, as well as the performed surgical procedures, which included an adenoidectomy or/and a tonsillectomy. The ENT clinical assessment used the drug-induced sleep endoscopy (DISE) only in particular cases where the level of airway obstruction was difficult to be appreciated on a clinical basis.

Adherence to treatment was evaluated by analyzing the parameters of the machine’s data logging. Good adherence was considered to be achieved in cases where the NIV time was higher than 4 h per night. Quantitative absolute and relative data were expressed as a median and interquartile range (IQR). The statistical analyses were performed using the Microsoft Excel software package, version 16.16.14.

## 3. Results

The demographic data and the distribution of diagnosed disorders in the study population are reported in Table 1. Briefly, this distinct study population included 108 children that were identified with neuromuscular diseases or complex genetic conditions, with 65 boys (60.2%) and 43 girls (39.8%).

Neuromuscular disorders were detected in 78 patients (72.2%) and other multiple congenital anomalies were detected in 30 patients (27.8%). Obesity was observed in 23 patients (21.3%) and 43 subjects (39.8%) had scoliosis. Some subgroups from our study included a small number of cases that were part of rarer genetic disorders.

OSAS was detected in 87 patients (80.5%) with different prevalence rates among various groups of genetic disorders in the study population, as shown in Table 2.

The respiratory parameters recorded using PSG are presented in Table 3. The median of obstructive apnea–hypopnea index (AHI) was 7.7 events/h with IQR = 5–14.2 events/h; the highest AHI = 117.5 events/h was observed in a patient with achondroplasia. The lowest SatO_2_ in patients with OSAS was 60% in a Crouzon disease patient. Sixty-two patients (71.3%) had SatO_2_ < 90% and 13 patients (15%) had hypoventilation. The highest TcCO_2_ of 78 mmHg was recorded in a neuromuscular patient.

As reported in Table 4, otorhinolaryngological surgery was performed n 15 patients (17.2%). Thirty-five patients (40.2%) started NIV, where 32 started BiPAP (36.8%) and 3 started CPAP (3.4%).

In the study population, 93.8% of the patients that were treated using BiPAP had neuromuscular disorders, while among the patients who used a CPAP therapeutic approach, two had Prader–Willi syndrome and one had achondroplasia. From the group with neuromuscular disorders, 40 patients (51.3%) had associated scoliosis. Three patients started NIV during a respiratory upper airway distress episode and hence, without a previous polysomnographic study, all other patients performed a formal sleep study prior to the implementation of the ventilatory support. Five patients were referred for another management option: neurosurgery for achondroplasia and Arnold–Chiari syndrome and ocular–orbital reconstruction for a patient with Crouzon disease.

The PSG parameters recorded at the post-therapeutic follow-up, which are presented in Table 5, confirmed the success of the treatments performed.

Minor complications of NIV were recorded in five (14.3%) patients as local skin irritation. All patients (100%) with sustained NIV had clinical improvement, as reported in the clinical files at the end of the sleep monitoring/titration process.

Although confirmed as having OSAS, 37 patients (42.5%) did not accept any therapeutic proposal and did not return for clinical follow-up. The reasons for minimal compliance are beyond the scope of this paper, but we suggest that multiple social and psychological factors contributed to this result.

For some patients, a specific therapeutic approach was advised: neurosurgery for achondroplasia and Arnold–Chiari syndrome and ocular–orbital reconstruction for a patient with Crouzon disease. Those cases did not come for follow-up either.

## 4. Discussion

The results of the present study showed that OSAS is a pathological condition with a significantly high prevalence in a selected population of children with neuromuscular, skeletal, genetic syndromes, and/or craniofacial abnormalities. The high prevalence of OSAS reported in this study (80.5%) is consistent with previously published studies for different populations and highlights the increased risk of OSAS among children with complex genetic disorders [17,21,23,26] compared with a much lower value of the prevalence of sleep-related breathing disorders (9.73%) in a general pediatric population [27]. Polysomnography played a key role in identifying OSAS in our pediatric population [22].

The comparative analysis of the results of the present study indicated a prevalence of 69.2% of OSAS in children with neuromuscular pathology in our group, which is a significantly high value that confirmed the trend published by other authors that report prevalences of over 40% [17,26,28,29,30,31]. In particular, OSAS was present in our study in 92% of children with Duchenne muscular dystrophy; this is a higher prevalence than that found in most previous studies, which ranged from 31 to 63% [32,33]. Our work identified OSAS in all children diagnosed with achondroplasia (100%); this is a result in disagreement with those published in the literature, which mentions prevalences between 54 and 59% for achondroplasia cases [17,26,28,29,30,31]. This discrepancy may be due to the small number of cases of the pediatric population enrolled in our study.

In cases of children with complex abnormalities, the prevalence of OSAS observed in our study is in line with the trend of values published by other authors, but with a higher incidence for all studied diseases. We identified OSAS in 100% of children with Crouzon syndrome, a higher value than the published ones, which are between 74 and 77% [34,35]; in children with Prader–Willi syndrome, we recorded an OSAS prevalence of 94.7%. Results published by other researchers show values between 79.9 and 92.9% [29,30]; we recorded an OSAS prevalence of 80% in cases with Arnold–Chiari syndrome, which is also higher than the published values of 60 to 72% reported by other authors [36,37,38].

A significantly higher prevalence of OSAS, both in our particular study group and in other previous publications, suggests a possible occurrence of neurocognitive complications on long-term evolutions. Some literature reports have demonstrated an abnormal central nervous system development in children with obstructive sleep apnea, followed by degradation in their quality of life [39,40,41]. This is why investigating sleep-disordered breathing in children with genetic diseases is important for delineating secondary neurocognitive deficits from their main associated pathology [17]. As a consequence, the recommendations for the proper diagnosis and management of OSAS come as a priority for children with complex neurological and genetic conditions [42].

In our group of patients, the association of scoliosis in 51.3% of children with neuromuscular disorders worsened the health condition of these children. Scoliosis was found to be associated with hypopnea or apnea with decreased oxygen saturations and hypoventilation, especially during rapid eye movement (REM) sleep [43]. It is also a risk factor for disease progression to daytime respiratory failure [4].

In a population of children with genetic abnormalities, the instabilities in the ventilatory control system can generate temporary arrest of the respiratory drive, hence the term central sleep apnea, which is also a consequence of the obstruction of the airway that triggers an elevated chemoreceptor sensitivity [44]. Both clinical forms, namely, mixed-dominant apnea and obstructive-dominant sleep apnea, could be met in a group of children with genetic disorders, but the mixed apnea could make the therapeutic approach more challenging [45].

In patients with neuromuscular diseases, NIV is the standard approach for sleep-disordered breathing because of specific characteristics, such as pharyngeal neuropathy or weakness, macroglossia, scoliosis, or small lung volumes. In our study, the use of CPAP and BiPAP has shown a complete control of sleep-disordered breathing, relieving the symptoms of OSAS. These results were achieved via careful monitoring, with manual titration of the positive airway pressure (PAP) that was conducted in 91.4% of the patients. PSG allowed us to produce accurate recordings during the NIV management. It is noteworthy that the non-invasive ventilation can also trigger specific sleep-disordered breathing events, such as air leaks, patient–ventilator asynchrony, and central sleep apnea [24]. After the therapy by CPAP, the mean value for AHI in the group in our study was 3 events/h, significantly higher than other studies for a special pediatric population that show values of 1.1 events/h [45], although the symptoms of our patients were totally controlled.

In the therapeutic approach taken for our patients, ENT surgery was used in those cases with obvious enlargement and obstructing pharyngeal lymphoid tissues. As a result, 73% of ENT surgeries were performed in patients with skeletal disorders and complex abnormalities (60% of our Prader–Willi patients), underlining the connection and summation influence of craniofacial anomalies with the obstruction of the upper airways in these patients.

Analyzing the PSG posttreatment parameters, the children from the group who benefited from ENT surgery showed a follow-up mean AHI of 1.33 events/h, which is a result that is consistent with data published by other studies on special pediatric populations that report values between 1.1 to 1.6 events/h in groups of patients with adenotonsillectomy [46].

In the end, multiple factors are associated with producing more severe diseases in children with genetic disorders. Allergic rhinitis should be medically controlled as an adjuvant measure, as rhinitis can also interfere with NIV compliance, with worsening nasal obstruction and mucosal dryness being frequent side effects that are also associated with NIV [21].

Clinical improvement should be monitored by performing follow-up sleep studies. Compliance with treatment is also an important issue in the assessment of NIV in children since it is closely related to the parental environment and other factors: child acceptance of the mask and early detection of possible skin lesions [21].

The low compliance with treatment recommendations was present in our study group in a large number of children (42.5%) with confirmed OSAS who did not accept the proposed treatment solutions and who did not continue follow-up monitoring of the disease’s evolution. We can explain this fact only in terms of the difficult access to health facilities of the medical system or by educational deficiencies of the parents or their lack of understanding of the long-term evolution of their child’s disease.

The gold standard for OSAS diagnosis remains PSG examination. However, in areas where PSG is not readily available, overnight pulse oximetry with continuous CO_2_ monitoring can also be used to record nighttime gas exchanges. Limited access to polysomnographic sleep studies should not delay these patients’ access to effective management plans [4]. A recent study demonstrated that nocturnal PSG contains more information about breathing than respiratory parameters alone, for example, snoring, mouth-breathing, and flow limitation [46]. These symptoms can also indicate a disturbance of breathing and occur earlier than apnea/hypopnea events associated with a decrease in oxygen saturation by 3% or more [46].

As in all retrospective studies, our research may have limitations, such as the availability of all categories of data for each patient, a limited number of patients with some specific pathologies, or the lack of a uniform therapeutic approach, but the results may guide the design of future improved studies.

## 5. Conclusions

The results of the present study suggest, in agreement with the literature data, that OSAS is a serious and frequent clinical condition for pediatric patients with complex genetic diseases, and plays a pivotal role in their disease symptomatology and worsens the quality of life of these children.

We showed that in genetic diseases, sleep-disordered breathing should not be considered an incidental complication. Because clinical symptoms cannot be used as predictors or markers for OSAS or nocturnal hypoventilation [21], the screening of overnight gas exchanges, at least to detect nocturnal hypoxemia or hypercapnia, with or without a complete sleep study if possible, should be a priority in all children with neuromuscular or genetic diseases. Detecting and treating sleep-disordered breathing in these patients may improve their already impaired quality of life [21].

Due to cognitive necessities and the rapid growth processes of children, the early diagnosis and treatment of OSAS is a priority for children with complex genetic conditions and requires a holistic understanding of the diagnosis, technology involved in diagnosis and therapy, prognoses, and long-term care. Our study strengthened the evidence for a multidisciplinary approach toward obstructing sleep apnea syndrome in children with neuromuscular or complex genetic disorders.

## Figures and Tables

**Table 1:** Demographic and clinical characteristics of the study population.

	Patients *n* = 108 (%) ^a^	Gender		Age at Time of First PSG (Years) Median [IQR]
		Female *n* = 43 (39.8%) ^a^	Male *n* = 65 (60.2%) ^a^	
**Neuromuscular pathology**	**78 (72.2%)**			
Spinal muscular atrophy (SMA)	**45**	**24**	**21**	-
SMA 1	12	4	8	7 mo [3,4,5,6,7,8,9,10]
SMA 2	25	14	11	8 [4,5,6,7,8,9,10,11,12,13]
SMA 3	8	6	2	11 [5,6,7,8,9,10,11,12]
Duchenne muscular dystrophy	**25**	**-**	**25**	14 [12,13,14,15,16]
Ulrich muscular dystrophy	**1**	**1**	**-**	11
Merosin deficient muscular dystrophy	**1**	**-**	1	5
Other myopathies	**6**	**2**	**4**	15 [12,13,14,15,16,17]
**Skeletal disorders**	**4 (3.7%)**			
Achondroplasia (ACH)	**3**	**3**	**-**	4 [1,2,3,4,5,6,7]
Marfan syndrome	**1**	**1**	**-**	17
**Complex abnormalities**	**26 (24.1%)**			
Craniosynostosis (Crouzon syndrome)	**2**	**1**	**1**	4 [3,4,5]
Prader–Willi syndrome (PWS)	**19**	**8**	**11**	4 [3,4,5,6,7,8,9,10,11]
Arnold–Chiari syndrome	**5**	**3**	**2**	11 [3,4,5,6,7,8,9,10,11]

PSG: polysomnography. IQR: interquartile range. ^a^ The percentage refers to the total number of patients in the study (N = 108).

**Table 2:** Prevalence of OSAS.

Genetic Disorder	Prevalence of OSAS
Neuromuscular diseases	69.2%
Prader–Willi syndrome	94.7%
Arnold–Chiari syndrome	80%
Achondroplasia	100%
Crouzon syndrome	100%

**Table 3:** Respiratory parameters recorded using PSG.

	Patients with OSAS *n* = 87 (80.5%)	Respiratory Characteristics in Polysomnography Median [IQR]			
		AHI (events/h)	Index > 3% (ODI/h)	SpO_2_ < 90% *n* (%) *	SatO_2_ Nadir (%)
**Neuromuscular disorders**	**59** **(67.8%)**			**41** **(69.4%)**	
Spinal muscular atrophy (SMA)	29	8.3 [3.1–15.9]	4.8 [2.9–1.7]	22	77
Duchenne muscular dystrophy	23	6.3 [6.3–9.5]	6.2 [2.9–0.5]	13	80
Ulrich muscular dystrophy	1	16	16.4	1	85
Merosin deficient muscular dystrophy	1	8.3	6.9	1	89
Other myopathies	5	13 [5.8–16.3]	15.3 [7.2–2.1]	4	82
**Skeletal disorders**	**4** **(4.6%)**			**4** **(100%)**	
Achondroplasia (ACH)	3	6.5 [3–117.5]	9.3 [5.3–100.3]	3	71
Marfan syndrome	1	8.4	6.1	1	84
**Complex abnormalities**	**24** **(27.5%)**			**17** **(70.8%)**	
Craniosynostosis (Crouzon syndrome)	2	27.5 [17.3–37.8]	26.7 [26.2–27.2]	2	60
Prader–Willi syndrome (PWS)	18	5.7 [3.7–9.6]	7.6 [5,6,7,8,9,10,11,12,13,14,15]	14	73
Arnold–Chiari syndrome	4	5.1 [1.3–9.1]	16.5 [15.1–17.5]	1	70

IQR: interquartile range. Obstructive apnea–hypopnea index (AHI) events/h. Oxygen desaturation index > 3% (ODI)/h. Number of patients with SpO_2_ < 90% is given as n (%), * percent from the group of disorders.

**Table 4:** The therapeutic approach and post-therapy follow-up in the study groups.

	Patients with OSAS	OSAS-Treated Patients *n* = 50			PSG after Therapy
	*n* = 87	ENT	CPAP	BiPAP	*n* = 29
		*n* = 15 (17.2%)	*n* = 3 (3.4%)	*n* = 32 (36.8%)	
**Neuromuscular disorders**	**59 (67.8%)**				
Spinal muscular atrophy (SMA)	29	3	-	14	10
Duchenne muscular dystrophy	23	1	-	11	6
Ulrich muscular dustrophy	1	-	-	1	1
Merosin deficient muscular dystrophy	1	-	-	1	1
Other myopathies	5	-	-	3	2
**Skeletal disorders**	**4 (4.6%)**				
Achondroplasia (ACH)	3	2	1	-	2
Marfan syndrome	1	1	-	1	1
**Complex abnormalities**	**24 (27.5%)**				
Craniosynostosis (Crouzon syndrome)	2	-	-	-	-
Prader–Willi syndrome (PWS)	18	8	2	1	6
Arnold–Chiari syndrome	4	-	-	-	-

**Table 5:** PSG parameters during the post-treatment follow-up.

Treatment	AHI (events/h)	SpO_2_ < 90%, *n* (%)	SatO_2_ Nadir (%)
ENT surgery	1.33 [1,2]	0	92
BiPAP	1.8 [1,2,3,4]	0	95
CPAP	3 [2,3,4]	0	95

## Data Availability

The data presented in this study are available on request from the corresponding author. The data are not publicly available due to ethical reasons.
